# Transcriptional Modulation of Penicillin-Binding Protein 1b, Outer Membrane Protein P2 and Efflux Pump (AcrAB-TolC) during Heat Stress Is Correlated to Enhanced Bactericidal Action of Imipenem on Non-typeable *Haemophilus influenzae*

**DOI:** 10.3389/fmicb.2017.02676

**Published:** 2018-01-12

**Authors:** Abdessalam Cherkaoui, Seydina M. Diene, Adrien Fischer, Stefano Leo, Patrice François, Jacques Schrenzel

**Affiliations:** ^1^Bacteriology Laboratory, Division of Laboratory Medicine, Department of Genetics and Laboratory Medicine, Geneva University Hospitals, Geneva, Switzerland; ^2^Genomic Research Laboratory, Division of Infectious Diseases, Geneva University Hospitals, Geneva, Switzerland

**Keywords:** heat stress, NTHi, RNA-seq, AcrAB-TolC, OmpP2, PBP1b, Bocillin-FL

## Abstract

**Objective:** The purpose of the present study was to investigate the penicillin binding proteins (PBPs), drug influx and efflux modulations during heat stress and their effects on the bactericidal action of imipenem on non-typeable *Haemophilus influenzae* (NTHi).

**Methods:** The two NTHi clinical isolates (GE47 and GE88, imipenem MICs by E-test > 32 μg/mL) examined in this study were collected at Geneva University Hospitals. The imipenem killing activity was assessed after incubation of the NTHi strains at either 37 or 42°C for 3 h with increasing concentrations of imipenem. The detection of PBPs was carried out by Bocillin-FL. Global transcriptional changes were monitored by RNA-seq after pre-incubation of bacterial cells at either 37 or 42°C, and the expression levels of relevant target genes were confirmed by qRT-PCR.

**Results:** Quantitation of NTHi viable cells after incubation with 0.25 μg/mL of imipenem for 3 h revealed more than a twofold decrease in GE47 and GE88 viable cells at 42°C as compared to 37°C. Transcriptome analysis showed that under heat stress conditions, there were 141 differentially expressed genes with a | log2(fold change)| > 1, including 67 up-regulated and 74 down-regulated genes. The expression levels of *ponB* (encoding PBP1b) and *acrR* (regulator of AcrAB-TolC efflux pump) were significantly increased at 42°C. In contrast, the transcript levels of *ompP2* (encoding the outer membrane protein P2) and *acrB gene* (encoding AcrB) were significantly lower under heat stress condition.

**Conclusion:** This study shows that the transcriptional modulation of *ponB, ompP2, acrR*, and *acrB* in the heat stress response is correlated to enhanced antimicrobial effects of imipenem on non-typeable *H. influenzae*.

## Introduction

Non-typeable *Haemophilus influenzae* (NTHi) commonly resides in the human nasopharynx, from which it can disseminate to other organs and cause two types of infections, differing in their epidemiology and severity. The non-invasive infections usually affect the upper respiratory tract, whereas the invasive infections include bacteremia, and meningitis (Tristram et al., 2007; Murphy et al., 2009; Langereis and de Jonge, 2015). The asymptomatic colonization of the human nasopharynx is considered as the first step in the pathogenesis. NTHi is able to persist in the respiratory tract and also to be responsible of recurrent infections (Leach et al., 1994). The treatment of invasive NTHi infection can be seriously affected by antibiotic resistance. For a long time, ampicillin (alone or in combination with chloramphenicol) was considered as the first-line antibiotic in the treatment of invasive *H. influenzae* (Cole et al., 1979). However, since the emergence of strains resistant to ampicillin, amoxicillin/clavulanic acid and second-generation cephalosporins (e.g., cefuroxime); extended-spectrum cephalosporins (e.g., ceftriaxone) have been extensively used. Therefore, finding alternatives to extended-spectrum cephalosporins for the treatment of invasive infections caused by resistant strains was considered important. This holds especially true in the context of the global spread of extended-spectrum-β-lactamases (ESBLs) in *Enterobacteriaceae*, as well as of the recent reports of ESBL (*bla*_TEM-15_) in the closely related *Haemophilus parainfluenzae* (Tristram et al., 2008). Nowadays carbapenems are largely used as an alternative to extended-spectrum cephalosporins for initial empirical treatment of severe infections until definitive microbiological analysis results are available. A steady and worrying increase in the incidence of invasive infections caused by NTHi associated to the global spread of resistant strains (Whittaker et al., 2017) highlights the importance to identify potential new antibiotic targets, or to enhance the activity of existing antibiotics. Furthermore, our previous data imply that the resistance to imipenem in NTHi should be seriously considered. Two β-lactams resistance mechanisms have been largely reported in *H. influenzae*. One requires TEM-1 or ROB-1 β-lactamases. The other one requires decreased β-lactams affinity for penicillin binding protein (PBP) 3. Nowadays, it is noticeable that the functions of PBPs are multiple and may differ according to growth conditions and physiological status. In addition, as it has been shown previously, β-lactams resistance in *H. influenzae* is often multi-factorial (Tristram et al., 2007; Cherkaoui et al., 2016).

In their hosts, NTHi must continuously cope with a broad spectrum of stress factors such as elevated temperatures, some nutrient limitation, host defense mechanisms, and antibiotics effects. Depending on their proprieties, stress triggers different highly regulated adaptive responses that do not only preserve bacteria from the environmental changes, but can besides lead to bacterial transformations that affect their susceptibility to antimicrobials (Poole, 2012). In response to heat stress conditions, bacteria induce many proteins called heat-shock proteins (HSPs). These proteins encompass chaperones and proteases that are important for overcoming the imbalance of protein homeostasis caused by heat stress. The importance of HSPs within the proteome can vary substantially from species to species. For instance, in *Escherichia coli* the level of HSPs decreases rapidly once the stress disappears. In contrast, in *Streptococcus pneumoniae*, the HSPs induced by heat stress remain detectable 1 h after the re-establishment of normal conditions (Tran et al., 2011). Regarding NTHi, there are only scarce studies on the function of stress-induced proteins, and the regulation of gene expression in response to heat stress. To further investigate relationships between β-lactams and PBPs, studies should consider temperature-sensitive.

To gain insights into the effect of heat stress on imipenem resistance in *H. influenzae*, we measured the interaction between controlled heat stress and imipenem resistance in NTHi by four mutually supportive approaches: (i) we simultaneously measured growth and cell viability at either 37 or 42°C in NTHi cells exposed to increasing concentrations of imipenem; (ii) we investigated PBPs by using Bocillin-FL and bacterial cells growth at either 37 or 42°C; (iii) we monitored transcriptome changes by RNA-seq after pre-incubation of bacterial cells at either 37 or 42°C; and (iv) we confirmed by real-time quantitative reverse transcription-PCR (qRT-PCR) the transcriptional changes of different key genes (*ponA, ponB, pbp2, ftsI, acrR, acrB, ompP2*) that can be implicated in the resistance of *H. influenzae* to β-lactam antibiotics.

## Materials and Methods

### Bacterial Strains

**FIGURE 1:**
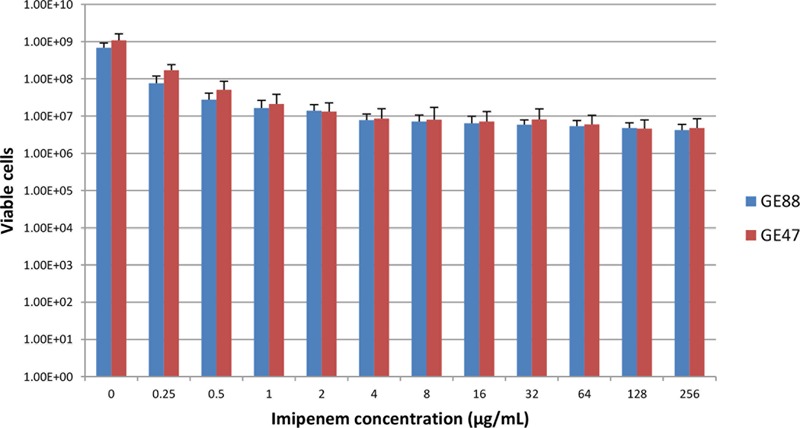
Viable NTHi cells after growth at 37°C in the presence of increasing concentrations of imipenem. Data are presented as means ± SD of 8 independent biological replicates.

The two highly imipenem resistant non-typeable *H. influenzae* strains (GE47 and GE88) examined in this study were taken from a collection of 46 NTHi strains previously studied (Cherkaoui et al., 2016). In more detail, GE47 and GE88 were grown in the presence of high concentrations of imipenem (**Figure 1**). Using E-test assay (bioMérieux), the imipenem MIC was greater than 32 μg/mL for the both strains (Supplementary Figure S1) and neither of them produces a β-lactamase, assessed by the chromogenic cephalosporin assay using a nitrocefin disk (Becton Dickinson) and the hydrolysis of penicillin G in the API *Neisseriae–Haemophilus* system (bioMérieux). Furthermore, these strains present amino acid substitutions in PBP3, PBP4, and AcrR (Supplementary Table S1). Susceptibility profiles of GE47 and GE88 strains to 14 antibiotics are reported in Supplementary Table S2. Finally, the two strains were isolated from the sputum of two 75 year-old patients.

The reference strain *H. influenzae* Rd KW20 was also used for the preparation of membrane proteins-enriched fraction used for the detection of PBPs.

### Growth and Viability of NTHi Cells at either 37 or 42°C

The inoculum suspension (50 mL) was prepared by picking several colonies from an overnight culture at 37°C on chocolate agar plates and suspending the colonies in Brain Heart Infusion (BHI) broth supplemented with NAD 2 μg/mL and hemin 10 μg/mL (sBHI) to a density of 0.5 McFarland. This inoculum suspension was transferred into two sets of 10 vials, each containing 2 mL of that inoculum suspension. One set was then incubated at 37°C and the other at 42°C. The optical densities (OD_600_) of 1 mL aliquots were plotted against time; and the amount of viable cells was determined at selected time points by plating culture dilutions onto chocolate agar plates, followed by overnight incubation at 37°C, and viable cell counting. The longest incubation time at 42°C that did not significantly affect cell growth was 3 h (Supplementary Figure S2). Therefore this incubation time was used in all following experiments. All culture incubations were carried out in the presence of 5% CO_2._

### Killing Activity of Imipenem on NTHi Cells at either 37 or 42°C

The killing activity of imipenem on NTHi cells was assessed after 3 h incubation at either 37 or 42°C with increasing concentrations of imipenem ranging from 0 to 256 μg/mL, following the procedure depicted in Supplementary Figure S3. The incubation of NTHi cells during 3 h at either 37 or 42°C with 0 μg/mL of imipenem was considered as a control condition in each experiment. The assay was performed in eight independent biological replicates. The number of viable cells was determined by plating culture dilutions on chocolate agar plates followed by overnight incubation at 37°C, and viable cell counting. The amount of GE47 and GE88 viable cells at either 37 or 42°C after incubation for 3 h with increasing concentrations of imipenem (range: 0.25–256 μg/mL), were normalized based on their amount of viable cells in a control condition. The data are expressed as a percentage of viable cells relative to the control condition for the different concentrations of imipenem. A paired Student’s *t*-test was performed with GraphPad Prism 6 software (GraphPad Software, La Jolla, CA, United States) and comparisons were considered statistically significant when associated *p*-values were < 0.05.

### Detection of Penicillin Binding Proteins

The detection of PBPs in NTHi was carried out by the non-radioactive synthetic fluorescent penicillin Bocillin-FL (Zhao et al., 1999). GE47, GE88 and the reference strain *H. influenzae* Rd KW20 were used for the preparation of membranes for the detection of PBPs by following the procedures described previously, with a few modifications (Morikawa et al., 2004; Asli et al., 2016). The overnight cultures (4 mL each) were inoculated into 400 mL of fresh *Haemophilus* Test Medium broth (HTM broth). Cell cultures were grown for 3 h at either 37 or 42°C, and harvested by centrifugation at 6,000 × *g* for 15 min. The cells were washed once with 67 mM potassium phosphate (pH 7.0), and re-suspended in the same buffer. Cells were treated with 3 μg/mL of protease inhibitor cocktail (Sigma-Aldrich), DNase (6 μg/mL), RNase (6 μg/mL), and lysozyme (400 μg/mL). After 30 min of treatment, cells were disrupted with Bioruptor^®^ Pico sonication device (Europe Diagenode SA/Seraing, Belgium). The resulting cell lysates were centrifuged at 15,000 × *g* for 30 min. The supernatant fractions were collected and centrifuged at 136,000 × *g* for 30 min. The pellets were collected, washed once, and re-suspended in the same phosphate buffer. The resulting suspensions were considered as membrane preparations and were used for the fluorescent Bocillin-FL binding assays. Determination of protein concentration was performed using the Bio**-**Rad protein assay.

Cells were labeled at either 37 or 42°C for 30 min with a Bocillin-FL. The reaction mixtures (55 μL each), which contained 35 μL of each membrane preparation (∼200 μg of protein), 20 μL of 50 μM (final concentration) Bocillin-FL, were incubated at either 37 or 42°C for 30 min. Then, 4 μL of 10% sodium sarcosine, including 180 μg of penicillin G per mL, were added to the reaction mixture and the mixture was centrifuged at 10,000 × *g* for 30 min. Twenty microliters of each of the resulting supernatants were denatured with 20 μL of sodium dodecyl sulfate denaturing solution at 100°C for 3 min. Then, for the sodium dodecyl sulfate polyacrylamide gel electrophoresis analysis (10% polyacrylamide), 10 μL of each reaction mixture were used. After electrophoresis the gels were rinsed with water for 1 h. To visualize the labeled PBPs, the gels were directly scanned with the Gel Doc^TM^ XR+ System (Bio-Rad Laboratories). The fluorescence intensity of each band was quantified by using Image LAB^TM^ software (version 5.1; Bio-Rad).

### Transcriptome Analysis

The transcriptome analysis was assessed only for GE47 strain. This strain was chosen because the cell growth was higher than that of GE88 during the three first hours of incubation at 42°C. Global transcriptional changes were monitored by RNA-seq after pre-incubation of bacterial cells for 3 h at either 37 or 42°C. Experiments were performed in two independent biological replicates. Total RNA of GE47 was purified from bacterial pellets re-suspended in 1 mL of TRIzol (Invitrogen) with 0.1-mm glass/zirconia beads and disrupted in a Bead Beater apparatus. The RNA in the aqueous phase was precipitated with ethanol, rinsed several times and solubilised in 50 μL of diethylpyrocarbonate-treated water. The concentration and purity of total RNA were determined by measuring the absorbance at 230, 260, and 280 nm using a Nanodrop-8000 spectrophotometer (Thermo Fisher). RNA integrity was assessed using the RNA Nano 6000 Assay Kit of the Agilent Bioanalyzer 2100 system (Agilent Technologies). RNA libraries were generated using NEBNext Ultra Directional RNA Library Prep Kit for Illumina (NEB), and sequenced on an Illumina Hiseq 2500 platform. The raw sequence data were filtered by removing reads containing adapter, reads containing poly-N, and low-quality reads. The filtered reads were aligned against the genome of *H. influenzae* Rd KW20 (NC_000907.1).

RNA-Seq data were further analyzed for statistics in the software R v3.2.3 with the package edgeR v3.10.5 (Robinson et al., 2010) as described previously (Anders et al., 2013). Briefly, we filtered out 164 out of 1846 genes which had counts per million lower than 1 in at least two samples (as two was the minimum number of samples used for each condition). The reads mapping to the remnant 1682 genes were normalized by weighted trimmed mean of M-values method and modeled according to a negative binomial distribution. To detect differentially expressed genes, we eventually performed pairwise comparisons between the two temperatures with the exact test. *p*-Values were corrected for false discovery rate (FDR). RNA-seq data have been submitted to ArrayExpress^1^ with the accession number: E-MTAB-6237.

### Real-Time Quantitative Reverse Transcription-PCR (qRT-PCR)

To confirm the RNA-Seq results, the expression levels of eight target genes were determined by qRT-PCR using the primers listed in Supplementary Table S3. First-strand cDNA was synthesized from total RNA extracted after 3 h incubation at either 37 or 42°C using SuperScript II (Invitrogen) on batch of 250 ng of total RNA. The mRNA levels of target genes (*ponA, ponB, pbp2, ftsI, acrR, acrB*, and *ompP2*) extracted from the GE47 strain grown at either 37 or 42°C were normalized based on their ribosomal RNA small subunit methyltransferase H (*rsmH*, originally designated as *mraW*) transcript level, which were assayed in each round of qRT-PCR. Data were presented as means ± SD of 4 independent biological replicates. Pairwise comparisons and paired Student’s *t*-test were done with GraphPad Prism 6 software as described above.

## Results

### Killing Activity of Imipenem on NTHi Cells at either 37 or 42°C

**FIGURE 2:**
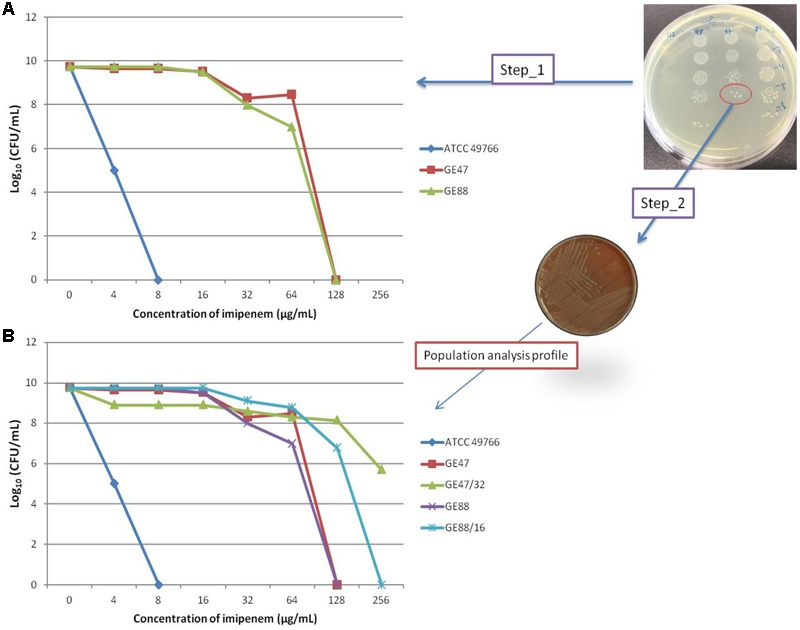
**(A)** Population analysis profiles of GE47 and GE88 strains as determined by microdilution plate counts. *Haemophilus influenzae* ATCC 49766 strain was used as a negative control for all experiments. **(B)** One colony of each of two NTHi strains was picked from HTM agar containing 16 or 32 μg of imipenem/mL and passaged in imipenem free HTM agar. After overnight growth, these cultures were named GE47/32 and GE88/16.

As depicted in **Figure 1**, the two NTHi strains analyzed in this study were highly imipenem resistant. Using E-test and macrodilution methods, the imipenem MIC was larger than 32 μg/mL for both strains. Moreover, the amount of viable NTHi cells after incubation at 37°C in the presence of increasing concentrations of imipenem revealed different according to imipenem concentration, suggesting the presence of heteropopulations. The population analysis profiles of GE47 and GE88 (**Figure 2A**) showed that with increasing concentrations of imipenem (range: 0–8 μg/mL), the subpopulations of cells did not differ considerably from each other in their imipenem susceptibility. To determine whether colonies of NTHi cells growing in the presence of high concentrations of imipenem were originating from populations of cells with a different resistant phenotypes, one colony of each of the two NTHi strains was picked from HTM agar containing 16 or 32 μg of imipenem/mL and passaged in imipenem free HTM agar. After overnight growth, these cultures named GE47/32 and GE88/16 were used as a starting cell suspension for further population analyses. Cultures of GE47/32 and GE88/16 were enriched in sub-populations resistant to 128 and 256 μg/mL of imipenem, respectively (**Figure 2B**).

The effect of heat stress on imipenem susceptibility was assessed on two highly imipenem resistant NTHi isolates (GE47, and GE88). As shown in Supplementary Figure S2, the amount of viable cells continued to increase significantly during the three first hours of incubation at 42°C, but the two isolates were differently affected by the heat stress. The GE47 cell growth was larger than that of GE88 during the three first hours of incubation at 42°C (after 3 h of incubation at 42°C, the number of viable cells was 1.21 × 10^9^ and 6.65 × 10^8^ for GE47 and GE88, respectively).

**FIGURE 3:**
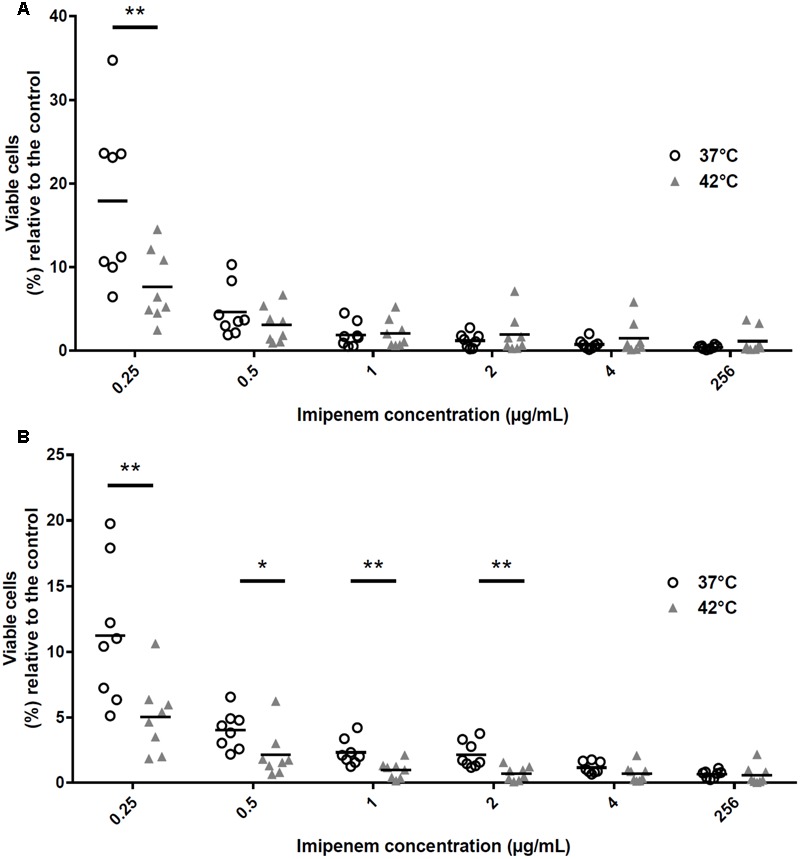
Percentage of viable NTHi cells relative to the control condition after exposition to increasing concentration of imipenem during 3 h at either 37 or 42°C. The amounts of GE47 and GE88 viable cells after incubation with increasing concentration of imipenem ranged from 0.25 to 256 μg/mL at either 37 or 42°C were normalized based on their amount of viable cells in a control condition (i.e., growth at either 37 or 42°C with 0 μg/mL of imipenem). No significant difference was observed on viable cells between 37 and 42°C when the bacterial cells were incubated with higher imipenem concentrations (>0.25 μg/mL for GE47 and >2.0 μg/mL for GE88) (Supplementary Figure S4). **(A)** GE47 strain (imipenem MIC by E-test = > 32 μg/mL); **(B)** GE88 strain (imipenem MIC by E-test = > 32 μg/mL). Experiments were performed in eight independent biological replicates. ^∗^*p* < 0.05, ^∗∗^*p* < 0.01 (paired Student’s *t*-test).

Based on their viability and growth levels at either 37 or 42°C without imipenem (control condition), quantitation of viable cells incubated with 0.25 μg/mL of imipenem revealed more than a twofold decrease in GE47 and GE88 viable cells at 42°C as compared to 37°C (**Figures 3A,B**). Unlike GE47, a significant difference was also observed for GE88 with imipenem concentrations ranging from 0.5 to 2.0 μg/mL (**Figure 3B**). However, no significant difference was observed on viable cells between 37 and 42°C when the bacterial cells were incubated with higher imipenem concentrations (>0.25 μg/mL for GE47 and >2.0 μg/mL for GE88) (Supplementary Figure S4), indicating that the increased imipenem susceptibility detected under heat stress conditions does not seem to be linked to imipenem heteroresistance. Importantly, for both strains, at 0.25 μg/mL of imipenem, the number of viable cells dropped substantially at 42°C as compared to 37°C, suggesting that heat stress potentiates bactericidal drug effects when the cells are incubated with low concentrations of imipenem.

### Binding of Bocillin-FL to Penicillin Binding Proteins

**FIGURE 4:**
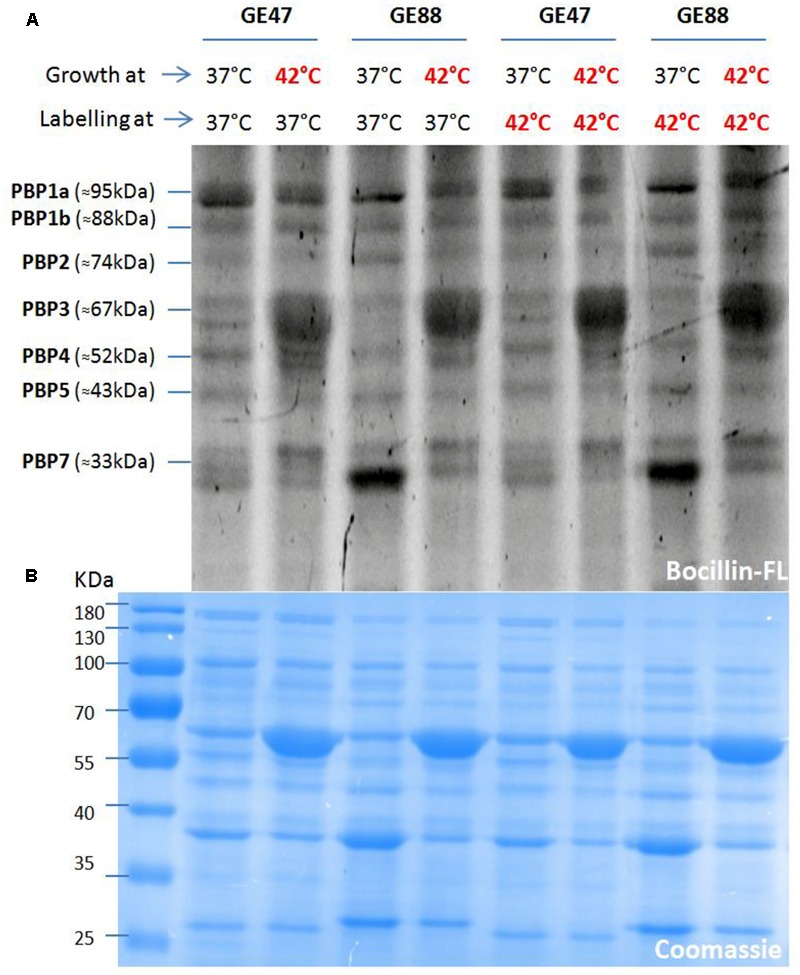
Binding of Bocillin-FL to penicillin binding proteins (PBPs) at two different growth and labeling temperatures (37 or 42°C). **(B)** Shows a stained image (Coomassie) of the top gel **(A)**, which indicates that for both strains (GE47 and GE88) the same amounts of membrane proteins were loaded in all the lanes.

**FIGURE 5:**
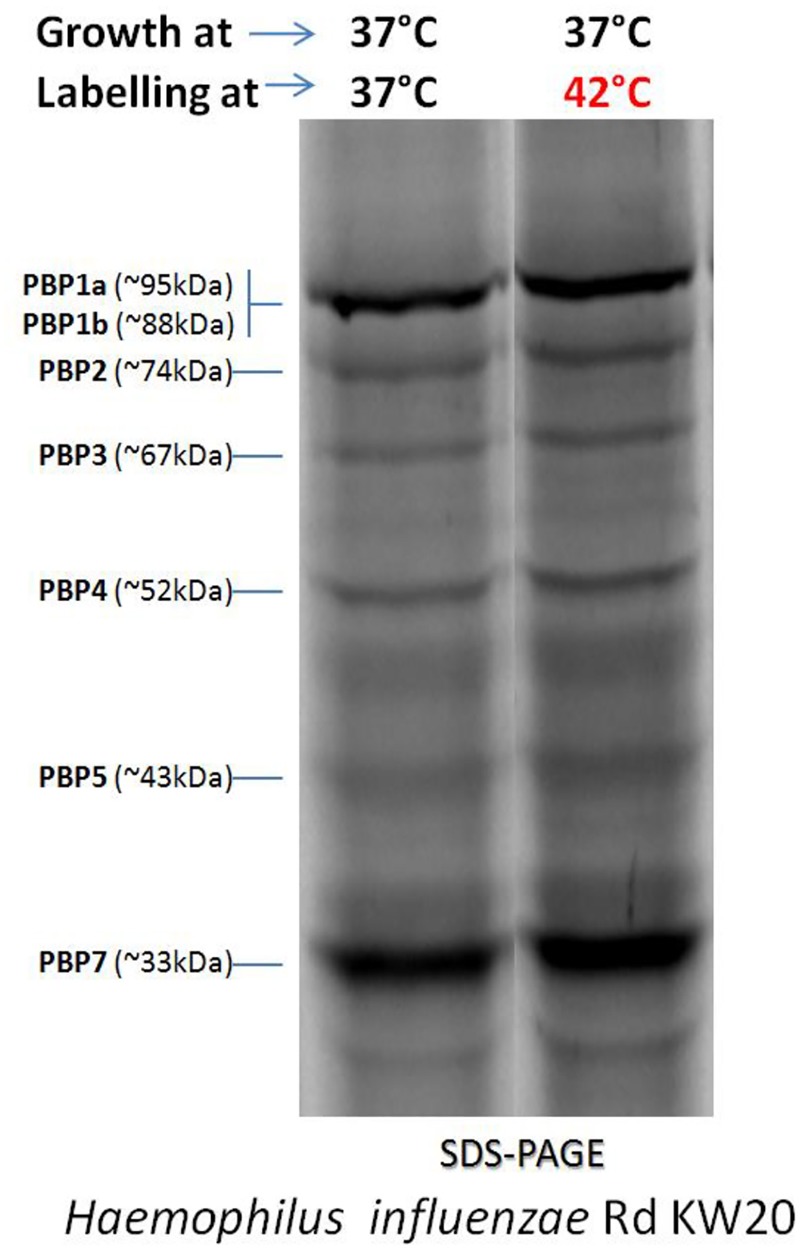
Bocillin-FL binding to PBPs of the reference *H. influenzae* strain Rd KW20 at two different labeling temperatures (37 or 42°C).

The Bocillin-FL binding to PBPs was investigated and performed at two different growth and labeling temperatures (37 or 42°C) for the two NTHi isolates. As depicted by Bocillin fluorescence measurements in SDS-PAGE gels (**Figure 4A**), the labeling at either 37 or 42°C when NTHi cells were grown at 42°C, showed for both strains (GE47 and GE88) a marked increase in fluorescence intensity corresponding to PBP3. However, under the same growth temperature, no significant difference in fluorescence intensity was observed after labeling at 37 or 42°C. Coomassie blue staining of SDS-PAGE gels showed that after growth at 42°C, the amount of proteins corresponding to PBP3 molecular weight was at least fourfold higher (**Figure 4B**). The NTHi strains studied here have altered PBP3 (Asp350Asn, Ala502val, Ala502Thr, Asn526Lys, combined with other mutations), which do not bind Bocillin-FL as well as the reference strain *H. influenzae* Rd KW20. Importantly, when labeled at 42°C, the Rd KW20 PBPs did not show significant difference in fluorescence intensities as compared to 37°C (**Figure 5**). Therefore, the increase in fluorescence intensity corresponding to PBP3 was not due to a change of the Bocillin-FL properties when incubated at 42°C.

Taking into account the specificity of Bocillin-FL binding affinity and the potential of the heat stress to induce the production of a large amount of proteins with molecular weights close to that of PBP3 (e.g., HSP70/DnaK and HSP60/GroEL, as confirmed by liquid chromatography tandem-mass spectrometry analysis, data not shown), it is likely that the observed fluorescence increase cannot be completely related to PBP3.

### Transcriptome Analysis of NTHi Strains Incubated at either 37 or 42°C

To investigate whether the use of two replicates was suitable for differential expression analysis, we generated a multidimensional scaling (MDS) plot to show the relationship existing between groups of pairs. In other words, a MDS plot was used as a variation of the usual principle coordinate plot where groups of samples are maximally separated based on their reciprocal differences. In more detail, we first filtered out genes that had an expression measured in counts per million higher than 1 in at least two out of all samples analyzed (we chose two since it is the minimum number of replicates present per condition). This procedure allowed us to exclude genes that had no counts in almost the totality of the samples. We therefore kept 1682 genes out of 1846. We then considered: (A) the top 500 genes with the largest standard deviation between samples; (B) the top 500 differentially expressed genes having the largest fold-changes between samples. We computed a pairwise-distance between samples for (A) and for (B) (Euclidean distance) and generated for both (A) and (B) a MDS plot (Supplementary Figure S5). In both cases, replicates clustered according to their own conditions and were separated from those of other temperatures. We conclude that the use of two replicates per condition was adequate here to perform differential expression analyses since intra-replicate variability was lower than inter-replicate variability. We would like to emphasize that the selection of differentially expressed genes has been performed by computing FDR-corrected *p*-values with the intent to limit as much as possible false positive results.

**FIGURE 6:**
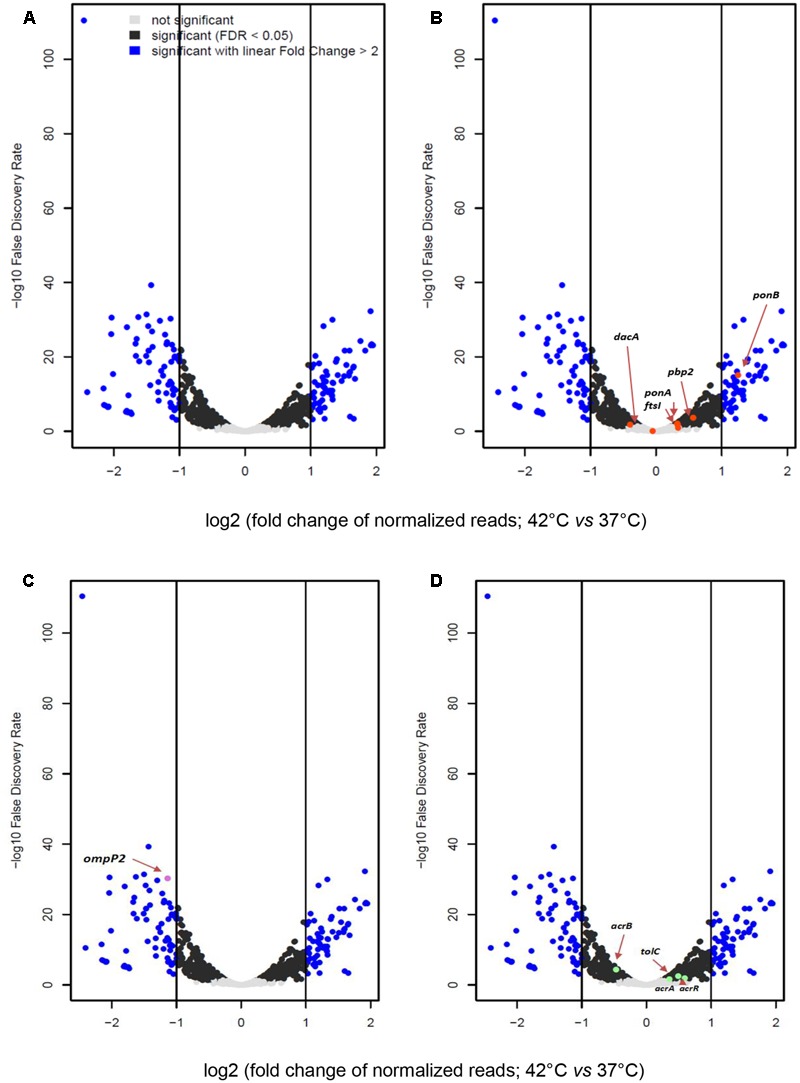
**(A)** Volcano plot reporting the –log10 false discovery rate (FDR) versus the log2 transformed fold change in read counts between 37 and 42°C. Each dot represents a given gene. Fold change is computed by considering the expression level at 42°C compared to 37°C, thereby all genes at the right of 0 of x-values are meant to be up-regulated at 42°C and those at the left of 0 of x-values are down-regulated at 42°C. Gray = genes that do not change in significantly expression; black = genes with expression values associated with FDR-corrected *p*-values; blue = genes expression values associated with *p*-value < 0.05 and linear fold change between 37 and 42°C higher than 2. **(B–D)** Same as **(A)** except that in **(B)** genes coding for PBPs are marked in red; in **(C)** outer membrane protein P2 gene is in violet, **(D)** efflux pumps (AcrAB-TolC) and the repressor AcrR-coding genes are depicted in green. *ponA* gene encoding PBP1a; *ponB* gene encoding PBP1b; *pbp2* gene encoding PBP2*; ftsI* gene encoding PBP3*; dacA* gene encoding PBP5.

**FIGURE 7:**
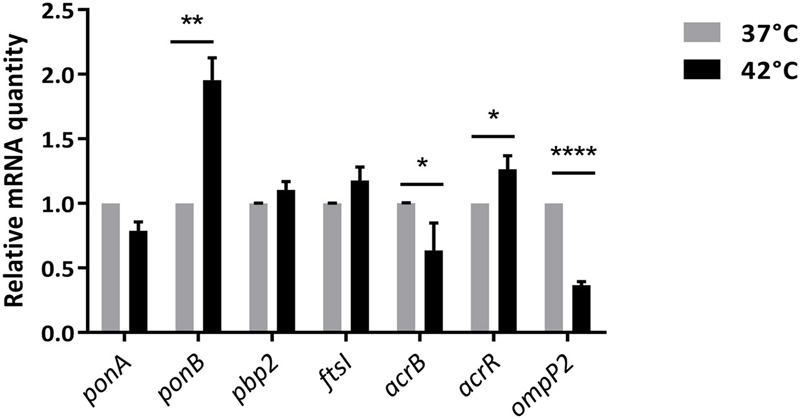
qRT-PCR of differentially expressed genes. The mRNA levels of target genes were normalized based on their ribosomal RNA small subunit methyltransferase H (RsmH) transcript level, which were assayed in each round of qRT-PCR. Data were presented as means ± SD of 4 independent biological replicates. ^∗^*p* < 0.05, ^∗∗^*p* < 0.01, ^∗∗∗^*p* < 0.001, ^∗∗∗∗^*p* < 0.0001 (paired Student’s *t*-test).

There were 141 differentially expressed genes with a |log2(fold change)| > 1 and a FDR < 0.05 under heat stress conditions, including 67 up-regulated and 74 down-regulated genes (Supplementary Table S4). A volcano plot was generated to visualize the distribution of these differentially expressed genes (**Figure 6**). The transcriptional changes of genes encoding for PBPs, outer membrane protein P2, efflux pumps (AcrAB-TolC) and the repressor AcrR, according to RNA-seq analysis were confirmed by qRT-PCR. The results were in good agreement with those from the RNA-seq analysis (**Figure 7** and **Table 1**). The expression levels at 42°C of *ponB* (encoding PBP1b) and *acrR* were significantly increased, but the expression of *ompP2* and *acrB* were significantly decreased.

**Table 1:** Summary statistics of RNA-seq profiles between 37 and 42°C of genes encoding in *H. influenza*e for different PBPs, OmpP2, AcrAB-TolC efflux pump and this negative regulator.

Gene	Annotation	Log2 (fold change)	LogCPM (logarithm of counts per million reads)	*p*-Value	False discovery rate (FDR)
*ponA*	Penicillin-binding protein 1a	0.320	10.333	0.004	0.010
*ponB*	Penicillin-binding protein 1b	1.250	9.000	0.000	0.000
*pbp2*	Penicillin-binding protein 2	0.563	9.422	0.000	0.000
*ftsl*	Penicillin-binding protein 3	0.329	10.162	0.007	0.016
*dacB*	Penicillin-binding protein 4	-0.050	7.812	0.714	0.785
*dacA*	Penicillin-binding protein 5	-0.396	8.766	0.007	0.017
*pbp7*	Penicillin-binding protein 7	0.337	6.681	0.068	0.118
*acrA*	Multidrug efflux system protein AcrA	0.354	9.604	0.012	0.027
*acrB*	Multidrug efflux system protein AcrB	-0.470	10.268	0.000	0.000
*tolC*	Membrane protein TolC	0.489	9.114	0.001	0.003
*acrR*	Regulator of the AcrAB-TolC efflux pump gene expression	0.589	7.438	0.004	0.011
*ompP2*	Outer membrane protein P2 (OmpP2)	-1.136	13.128	0.000	0.000

Coomassie blue staining of SDS-PAGE gels showed that after growth at 42°C, the amount of proteins corresponding to PBP3 molecular weight was at least fourfold higher. However, it was not correlated with the important increase in the expression level of *ftsI* gene in same condition (42°C). We postulate that the increase in the amount of the PBP3 may contribute to more stability of mRNA than increase in the expression of the *ftsI* gene.

Starting with our observation that the *ompP2* was down-regulated at 42°C but, strikingly, the exposition to heat stress enhanced NTHi susceptibility to low concentrations of imipenem, we investigated the expression levels of genes encoding different HSPs (SurA, DnaK, GroL, ClpB, and GroS) (Supplementary Figure S6A). All these proteins displayed increased transcript levels at 42 versus 37°C. In addition, no significant differences were observed in expression level of cluster of genes required in cell division and cell wall (*dcw*) biosynthesis (Supplementary Figure S6B), except for *fstQ, fstA*, and *ddlB*.

## Discussion

This study originated with the unexpected observation that highly imipenem resistant NTHi strains, although viable when exposed at 37°C to high concentration of imipenem (**Figure 1**), revealed more susceptible to lower concentrations of this antibiotic (0.25–2 μg/mL) when the cells were grown at 42°C. This raised the question of how does heat stress contribute to the active enhancement of imipenem susceptibility. Heat stress is one of the most significant environmental factors impacting bacterial physiology, which trigger adaptive and protective responses, notably by altering gene expression patterns. These major changes can affect cell physiology in different ways that can ultimately influence antimicrobial susceptibility profiles (Laport et al., 2001; Singh et al., 2007; Poole, 2012; Tomoyasu et al., 2012). Nowadays, a lot of information is available regarding the regulation of gene expression in response to heat stress in various bacterial species. However, there is currently no specific work dealing with the ability of NTHi cells to increase their antimicrobial susceptibility after heat stress. In this investigation, we observed that on the basis of their viability and growth levels at either 37 or 42°C without imipenem, the quantitation of viable cells pre-exposed to 0.25 μg/mL of imipenem revealed more than a twofold decrease in GE47 and GE88 viable cells at 42°C as compared to 37°C. This interesting observation raises many interrogations on the modulation of PBPs functions, cell wall structure and imipenem susceptibility by heat stress. As observed previously with *E. coli*, PBP1b interacts directly with PBP3 (Bertsche et al., 2006), which is required for the septal peptidoglycan synthesis (Nguyen-Disteche et al., 1998). B-lactams affect PBP1b or both, PBP1a and PBP1b, to rapidly lyse cells in *E. coli* (Yousif et al., 1985). Importantly, the inactivation of PBP2 or PBP3 by other target-specific β-lactam drugs causes different effects on bacteria cells. In more details, the inactivation of PBP2 results in the formation of spherical cells while the inhibition of septation and the occurrence of filamentous cells are induced by the inactivation of PBP3 (Tuomanen et al., 1986). Like in *E. coli*, the inactivation of PBP3 by target-specific β-lactams in *H. influenzae* leads to the formation of filamentous cells. Malouin and Bryan (1988) previously reported that *H. influenzae* cells grown at 42°C show filamentous cell formation. This growth aspect can explain a steady increase in the absorbance after 3 h at 42°C but a reduction in the overall viability of bacterial cells (Supplementary Figure S2). We previously observed that imipenem shows high affinity for PBP3 (IC_50_, 0.004 μg/mL) and PBP1b (IC_50_, 0.75 μg/mL) (Cherkaoui et al., 2016).

In a previous study it has been shown that *H. influenzae* displays a characteristic temperature-sensitive PBP3 with a reduced penicillin-binding activity at 42°C (Malouin et al., 1988). Taking into account the fact that in *E. coli*, the inhibition of PBP1b alone or combined with that of PBP1a by β-lactams causes the rapid lysis of bacteria cells, it is more than likely that an additional inhibitory effect linked to the temperature-sensitive PBP3 should be considered for the increased imipenem sensitivity of GE47 and GE88 strains grown at 42°C. Furthermore in *E. coli*, a mutation impacting the *ftsA* gene expression, which is a part of cluster of genes required in cell division and cell wall (*dcw*) biosynthesis, induces alterations in PBP3 phenotype at 42°C (Tormo et al., 1986). In GE47 strain, the *ftsA* was shown down-regulated at 42°C.

The observation that at 42°C, *H. influenzae* PBP1b and PBP3 showed different transcript levels suggests that the optimal activities of these PBPs were modulated by the physiological status of the bacteria cells. Modifications in PBP patterns like the reduction of PBP affinity to β-lactams, the decrease in the PBP transcript levels, or the presence of new PBPs, were reported in several other bacterial species. For example, *Enterococcus faecium* mutants that are PBP3 temperature-sensitive, do also overproduce PBP5. This allows these mutants to grow for 150 min at 42°C before cell lysis (Canepari et al., 1987; Fontana et al., 1994). Thus, the data reported in this study suggest that PBP1b, although not essential for imipenem resistance at 37°C, becomes important under heat stress conditions, yet without being able to strictly isolate the role of each molecular component, due to the compensatory effects of other PBPs.

Mechanisms affecting porins and efflux pumps enable bacteria cells to overcome the action of antibiotics, as they can restrict the interactions between the drug and its intracellular targets. Outer membrane protein P2 (OMP P2), representing the most abundant protein in the NTHi outer membrane, has porin activity and is considered as the target of bactericidal antibodies. The quality control of outer membrane proteins biogenesis and the structure of the bacterial membranes or cell wall appears negatively affected under heat stress conditions, thereby affecting the import or export fluxes of antibiotics through such bacterial membranes (McMahon et al., 2007; Ge et al., 2014). This suggests that the down-regulation of *ompP2* under heat stress conditions could be functionally involved in the increased imipenem killing observed with 0.25 μg/mL.

AcrAB-TolC belongs to the resistance-nodulation-division (RND) family of transporters. AcrAB is encoded by the *acr* operon that includes *acrA*, and *acrB* genes. The transcription of *acr* operon is negatively regulated by its repressor *acrR* (Dean et al., 2005). The combined increase of *acrR* and decrease of *acrB* expression levels, reveals that under heat stress, imipenem overcomes efflux-mediated multidrug resistance. These observations strongly imply that down-regulation of AcrAB-TolC is one of the major mechanisms accounting for enhanced NTHi susceptibility to imipenem under heat stress conditions.

Different chaperones have been reported in *H. influenzae*, including HSP70/DnaK and HSP60/GroEL and most of them are heat inducible (Hartmann and Lingwood, 1997). Major HSPs are implicated in the correct folding and assembly of proteins. In addition they are required in various cell processes (DNA replication, RNA transcription, bacterial growth). According to the duration and severity of the heat stress condition, the accumulation of misfolded proteins can lead to the death of the bacteria. Nonetheless, if the heat stress is not lethal, the bacterial cells become more tolerant against more severe stress conditions (Ellis and van der Vies, 1991). As depicted in Supplementary Figure S6, SurA, DnaK, GroL, ClpB, and GroS displayed increased transcript levels at 42°C, suggesting their roles in the growth of NTHi cells at such temperatures, again by the evidence of correlations but not formally from a mechanistic standpoint.

In NTHi, LytM factors are reported to have a direct effect on cell division by influencing the structure of the peptidoglycan (Ercoli et al., 2015). In the present study, no differences were observed in the transcription levels of LytM factors under heat stress.

## Conclusion

This study has identified the specific heat stress-regulated genes and processes involved in imipenem resistance. The growth of NTHi cells at 42°C rendered the organism unable to withstand a subsequent imipenem challenge as compared to NTHi cells grown at 37°C. The increased expression level of *ponB* (encoding PBP1b) shown in this study may reflect an increased physiological activity of this PBP toward imipenem. In addition, PBP1b could take over the function of the temperature-inactivated PBP3. Yet, this postulate still needs to be proven in future studies, since these elements are derived from correlations.

In this study we provided also evidence that the relationship between imipenem susceptibility and heat stress response depends largely on the down-regulation of OmpP2 and AcrAB-TolC. This suggests that bacterial regulatory networks may play a role in the control of the of the imipenem resistance phenotype, and in the enhancement of NTHi susceptibility to imipenem under heat stress conditions. Dissecting such pathways in further genetic studies might help identifying potential antibiotic targets.

## Author Contributions

AC: performed the experiments and drafted the manuscript. SD: carried out the transcriptome analysis and participated in drafting the manuscript. AF: carried out the statistical analysis of killing activity of imipenem. SL: carried out the transcriptome analysis. PF and JS: supervised the research and participated in drafting the manuscript. All authors read and approved the final manuscript.

## Conflict of Interest Statement

The authors declare that the research was conducted in the absence of any commercial or financial relationships that could be construed as a potential conflict of interest.

## Footnotes

1 https://www.ebi.ac.uk/arrayexpress/
