# Supplementary material for: Transcriptional Modulation of Penicillin-Binding Protein 1b, Outer Membrane Protein P2 and Efflux Pump (AcrAB-TolC) during Heat Stress Is Correlated to Enhanced Bactericidal Action of Imipenem on Non-typeable Haemophilus influenzae
Source: Front Microbiol. 2018 Jan 12;8:2676. doi: 10.3389/fmicb.2017.02676 (PMC5770572; doi:10.3389/fmicb.2017.02676)
Supplement: Supplementary file 1 [file Data_Sheet_1.pdf]

Transcriptional modulation of penicillin-binding protein 1b, outer membrane protein P2 and efflux pump (AcrAB-TolC) during heat stress is correlated to enhanced bactericidal action of imipenem on nontypeable *Haemophilus influenzae*

A. Cherkaoui[1], S. M. Diene[2], A. Fischer[1], S. Leo[2], P. François[2], J. Schrenzel[1,2]

[1]Bacteriology Laboratory, Department of Genetics and Laboratory Medicine, Geneva University Hospitals, 4 rue Gabrielle-Perret-Gentil, 1205 Geneva, Switzerland

[2]Genomic Research Laboratory, Division of Infectious Diseases, Geneva University Hospitals, 4 rue Gabrielle-Perret-Gentil, 1205 Geneva, Switzerland

Supplementary Materials

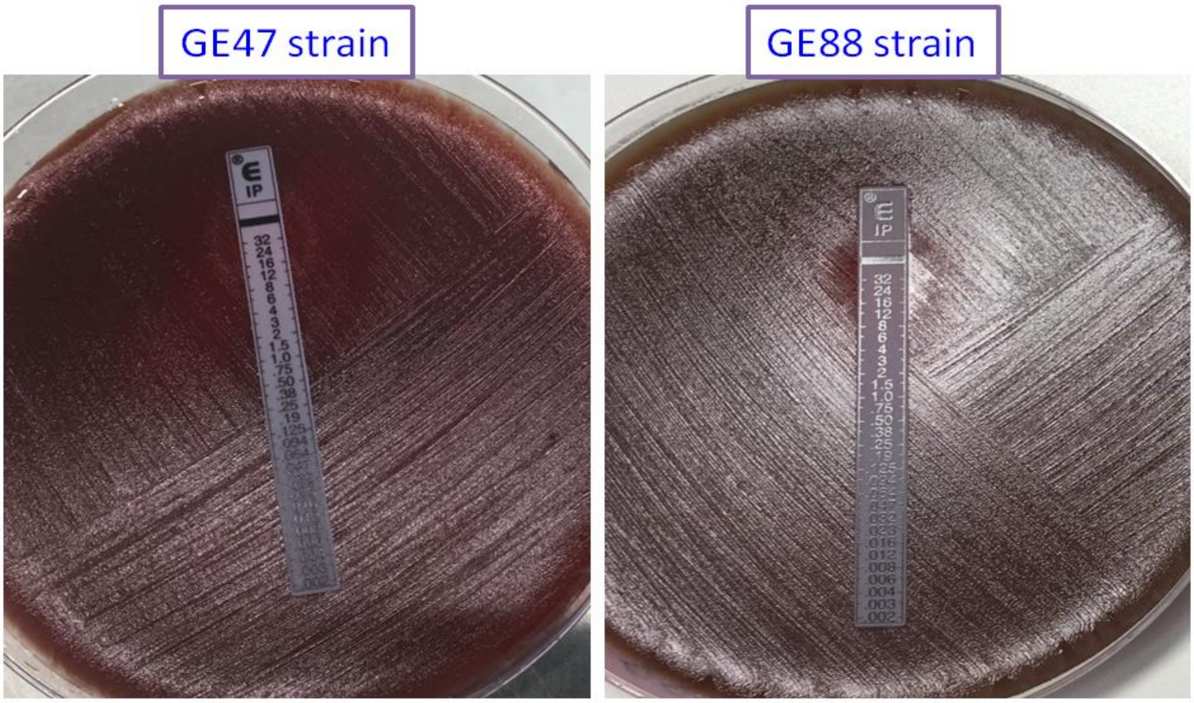

Supplementary Fig.S1: Imipenem MICs determination using E-test method

The imipenem MIC for both strains was greater than 32µg/mL

Group	Strain (n)	Ampicillin MIC range (µg/ml)	Imipenem MIC range (µg/ml)	Amino acid substitution for :																									
					Near *STVK motif					Close to SSN motif						Surrounding KTG motif													
	Rd KW20	0.064 - 0.094	0.25 - 0.38	Glu-141	Ser-273	Glu-274	Ser-311	Asp-350	Ser-357	Met-377	Ser-385	Leu-389	Ala-437	Ile-449	Gly-490	Ala-502	Val-511	Arg-517	Asn-526	Ala-530	Thr-532	Val-547	Asp-569	Ala-586	Ala-587	Asp-589	Thr-591	Ile-601	Glu-603
IIb	GE47	2	>32	.	.		.	Asn	.	.	.	.	.	.	.	Val	.	.	Lys	.	.		.	Pro	Val	Lys	Ala	Val	Asp
IIc	GE88	0.19	>32	Lys	.	Asp	.	Asn	.	.	.	.	.	.	.	Thr	.	.	Lys	.	.	Ile	Ser	.	.	.	.	.	Asn

Amino acid substitutions identified in the transpeptidase domain of the ***ftsI* gene** (encoding for PBP3)
*shows the position of the catalytic serine residue

Strain ID	Amino acid substitution for :					
Rd KW20	Asp-84	Asn-101	Asn-107	Ala-172	Glu-262	Gln-270
**GE88**	Gly	Ser	His	.	Ala	.
**GE47**	.	.	.	Ser	Ala	Lys

Amino acid substitutions identified in the transpeptidase domain of the ***dacB* gene** (encoding for PBP4)

Strain ID	Amino acid substitution for :			
Rd KW20	Leu-31	Ile-121	His-131	Gln-134
**GE47**	His	.	.	.
**GE88**	His	Val	Asp	Lys

Amino acid substitutions identified in the transpeptidase domain of the ***acrR* regulatory gene of the AcrAB-TolC efflux**

Supplementary Table S1: Amino acid substitutions in the *ftsI*, *dacB*, and *acrR* genes for two imipenem resistant nontypeable *H. influenzae* strains (GE47 and GE88)

Antibiotic	Strain ID	MIC µg/ml	EUCAST clinical breakpoints	CLSI clinical breakpoints
Imipenem	GE 47	>32	R	R
	GE88	>32	R	R
Ampicillin	GE 47	2	R	I
	GE88	0.19	S	S
Amoxicillin / clavulanic acid	GE 47	1.5	S	S
	GE88	0.125	S	S
Piperacillin/ tazobactam	GE 47	0.023		S
	GE88	0.016		S
* Cefuroxime	GE 47	3	R	S
	GE88	0.75	S	S
Cefotaxime	GE 47	0.047	S	S
	GE88	0.047	S	S
Ceftriaxone	GE 47	0.023	S	S
	GE88	0.032	S	S
Cefixime	GE 47	0.032	S	S
	GE88	0.047	S	S
Ceftobiprole	GE 47	0.064		
	GE88	0.064		
Ertapenem	GE 47	0.094	S	S
	GE88	0.25	S	S
** Meropenem	GE 47	0.19	S	S
	GE88	0.75	S	R
Levofloxacin	GE 47	0.023	S	S
	GE88	0.023	S	S
Co-trimoxazol	GE 47	4	R	R
	GE88	>32	R	R
Clarithromycine	GE 47	12	I	I
	GE88	24	I	I
Benzylpenicillin (screen) 1 unit	GE 47	11 mm	R	
	GE88	6 mm	R	
Ampicillin (2 µg)	GE 47	14 mm	R	
	GE88	6 mm	R	
Imipenem (10 µg)	GE 47	6 mm	R	R
	GE88	10 mm	R	R

Supplementary Table S2: Susceptibilities of GE47 and GE88 to 14 antibiotics

* EUCAST clinical breakpoints for intravenous drug administration
** EUCAST clinical breakpoints for infections other than meningitis

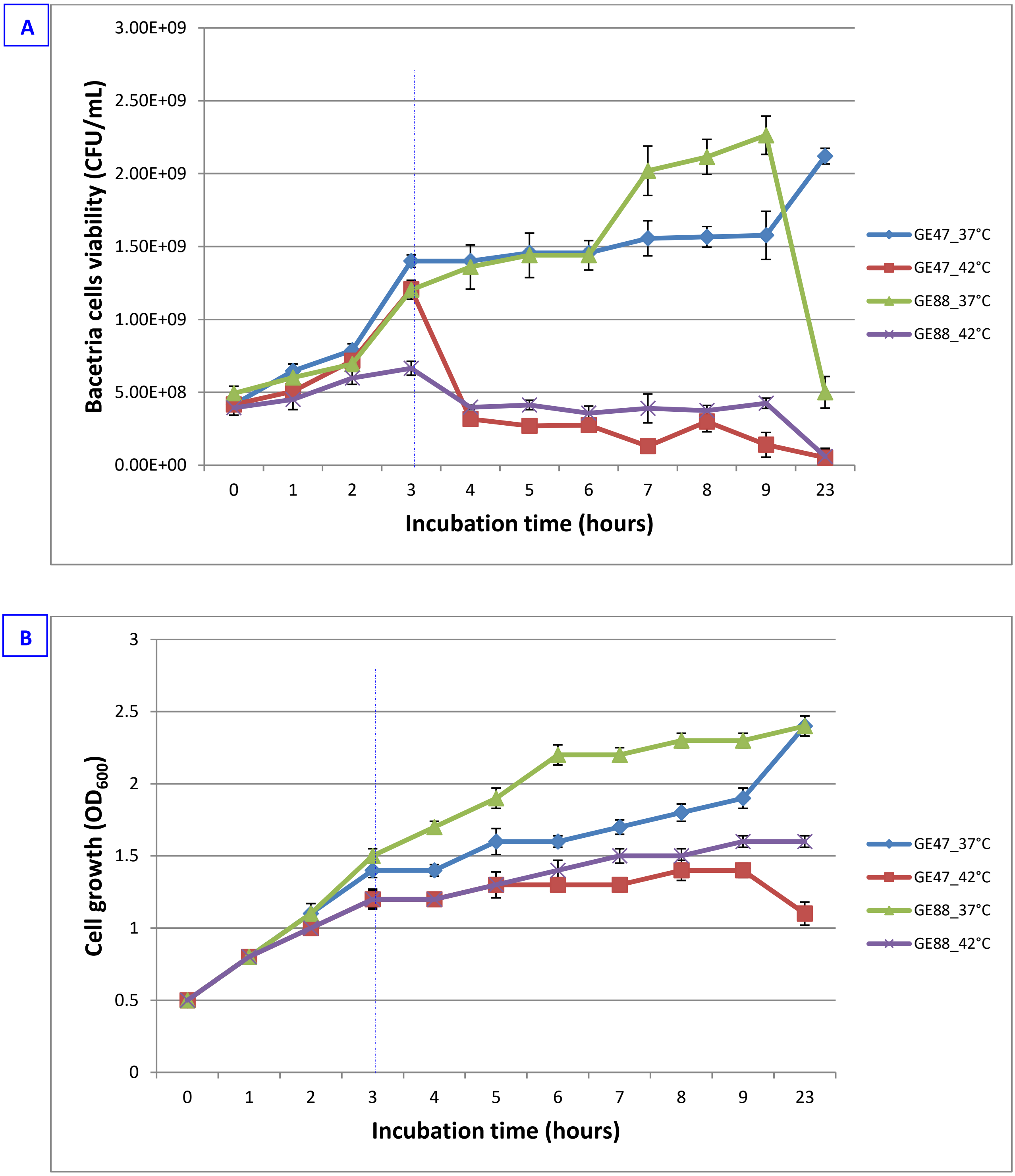

Supplementary Fig.S2: (**A**) Bacterial cell viability and (**B**) growth curves of GE47 and GE88 strains at either 37°C or 42°C in XV-supplemented brain heart infusion broth (sBHI).

The inoculum suspension was prepared by picking several colonies from an overnight growth on chocolate agar plates and suspending the colonies in sBHI to a McFarland 0.5 standards density. Inoculated sBHI media were incubated at either 37 or 42°C. Values represent the mean ± SD from 2 independent biological replicates.

The longer incubation time at 42°C that did not significantly affect cell growth was 3h; this incubation time was then used in all experiments.

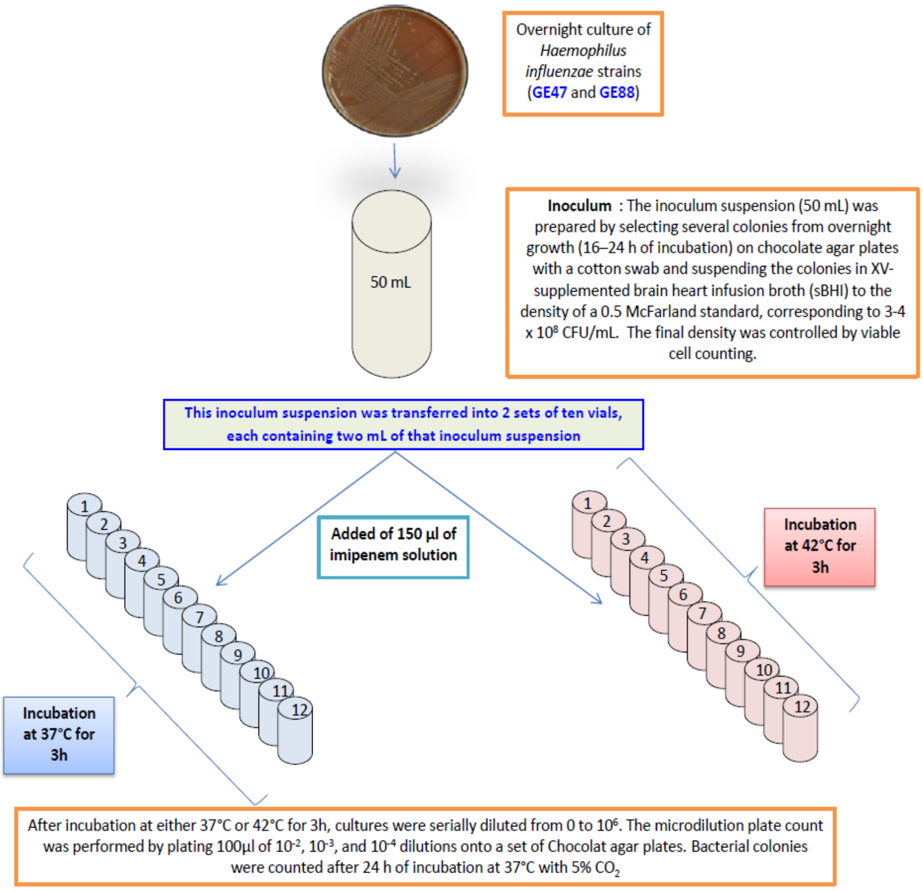

Tube ID	Imipenem final concentration
1	0 µg/mL
2	0.25 µg/mL
3	0.5 µg/mL
4	1 µg/mL
5	2 µg/mL
6	4 µg/mL
7	8 µg/mL
8	16 µg/mL
9	32 µg/mL
10	64 µg/mL
11	128 µg/mL
12	256 µg/mL

Supplementary Fig.S3: Assay procedure for viability measurement of NTHi cells after incubation with increasing concentrations of imipenem at either 37 or 42°C.

Target	Gene		Sequence	References
PBP1a	***ponA***	*Forward*	TCGGCGAGCAAATTTGGATT	
		Reverse	AAGCCACCGACCACTGCTTC	This study
		Probe	GCGTGCTAATGGGGAATGGCA	
PBP1b	***ponB***	*Forward*	CGGTACAGGGCGGAAGTACG	
		Reverse	CAAGGCTTCGTTGGCTTTGC	This study
		Probe	TTTATCGCGCGAACGCACCA	
PBP2	***pbp2***	*Forward*	GGTATGCCAACGGGGATTGA	
		Reverse	CCGAAATCGTATCGCCTTGC	This study
		Probe	CGGCTGCCAATATACCAACTCGAGA	
PBP3	***ftsI***	*Forward*	CCGCCAGTTATTGGGAAACG	
		Reverse	TTTACGCCGACACGGTAGCC	This study
		Probe	GCAATTAAAAATAAACGCGCAATGGTG	
AcrB	***acrB***	*Forward*	AGTTTCTTATCTGGTGCGACAGTTAC	
		Reverse	ATCTCGTTTTACCTGCGAAATGAC	This study
		Probe	TGTGGATGTGGATGGACGCGCTTA	
AcrR	***acrR***	*Forward*	GCGACAGATCGTTTAATGGCAAG	
		Reverse	GGTAAATCGTTCCTGCGGCTA	This study
		Probe	TGCTCAAACTTGCGAAAGAAGCAAA	
Omp2	***omp2***	*Forward*	CGTTGGTGCATTCGCAGCTT	
		Reverse	TCTGCGATAATGCTTAAACGACCA	This study
		Probe	CAGCAGCAAACGCAGCTGTTGT	
RsmH*	***rsmH***	*Forward*	GGGCAAAATTGACGGTATTTTG	
		Reverse	AAAACCACGTTCTGCTTCATCA	This study
		Probe	TTGATCTTGGTGTGTCTTCCCCTCAGC	

Supplementary Table S3: List of the primers designed and used in this study

*Ribosomal RNA small subunit methyltransferase H (originally designated as MraW).

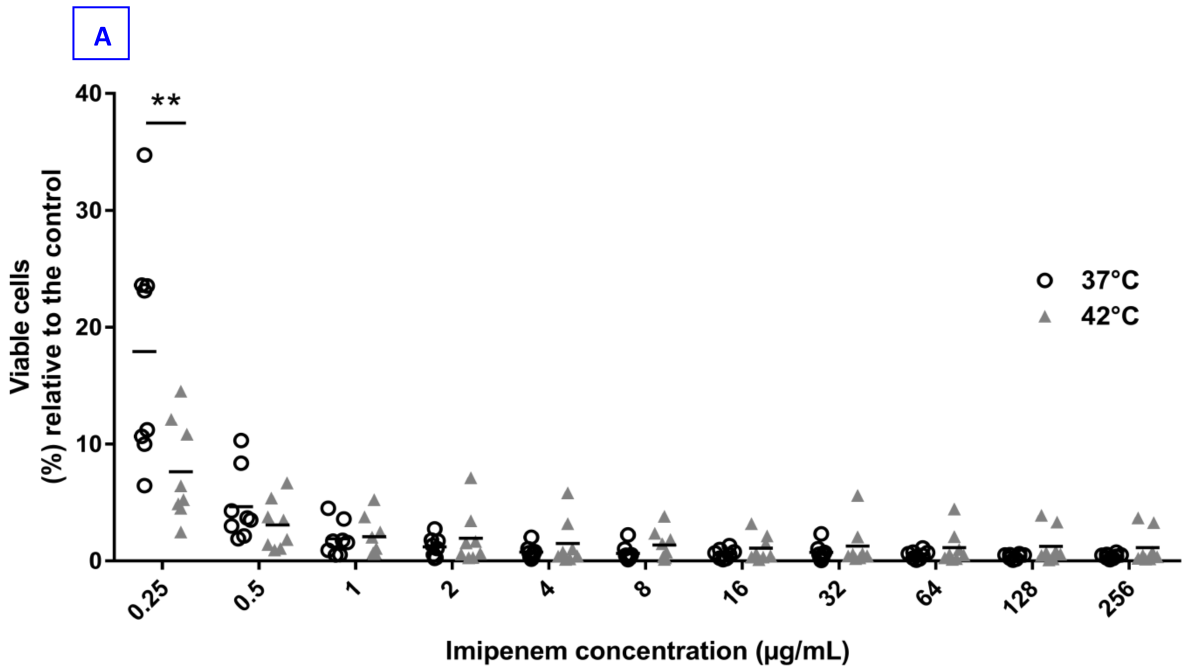

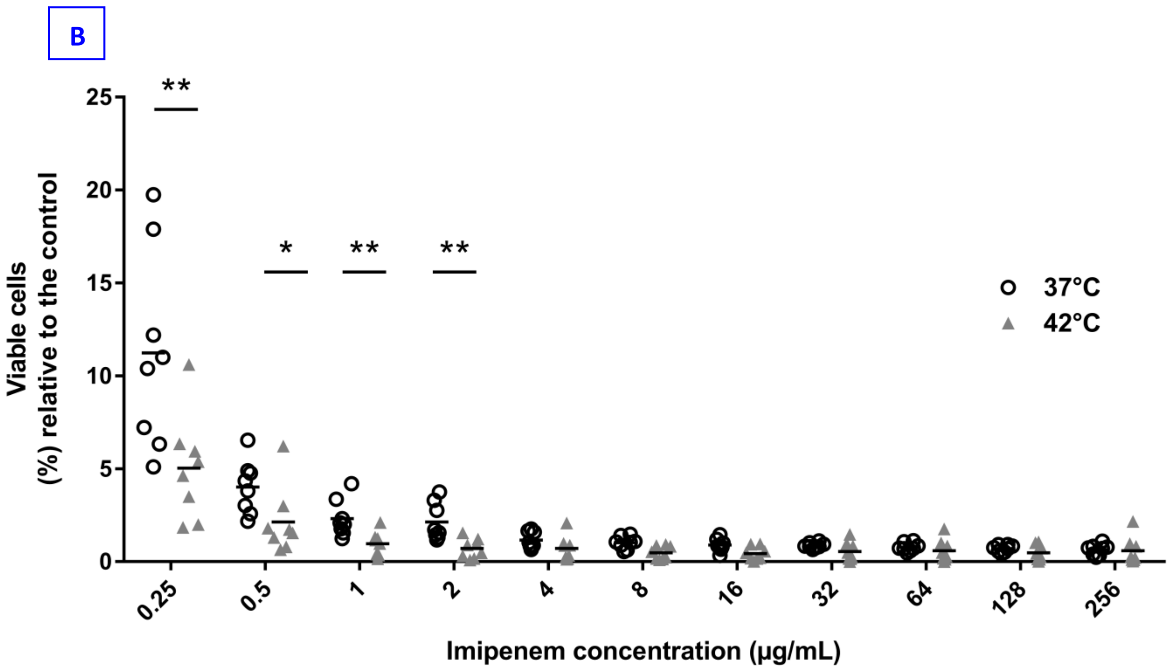

Supplementary Fig.S4: Percentage of viable NTHi cells relative to the control condition after exposition to increasing concentration of imipenem during 3h at either 37 or 42°C.

The amounts of GE47 and GE88 viable cells after incubation with increasing concentration of imipenem ranged from 0.25 to 256 µg/mL at either 37 or 42°C were normalized based on their amount of viable cells in a control condition (i.e. growth at either 37 or 42°C with 0µg/mL of imipenem).

(A): GE47 strain (imipenem MIC by E-test = >32 µg/mL)
(B): GE88 strain (imipenem MIC by E-test = >32 µg/mL)

Experiments were performed in 8 independent biological replicates.
* = p <0.05, ** =p <0.01 (paired Student's t test).

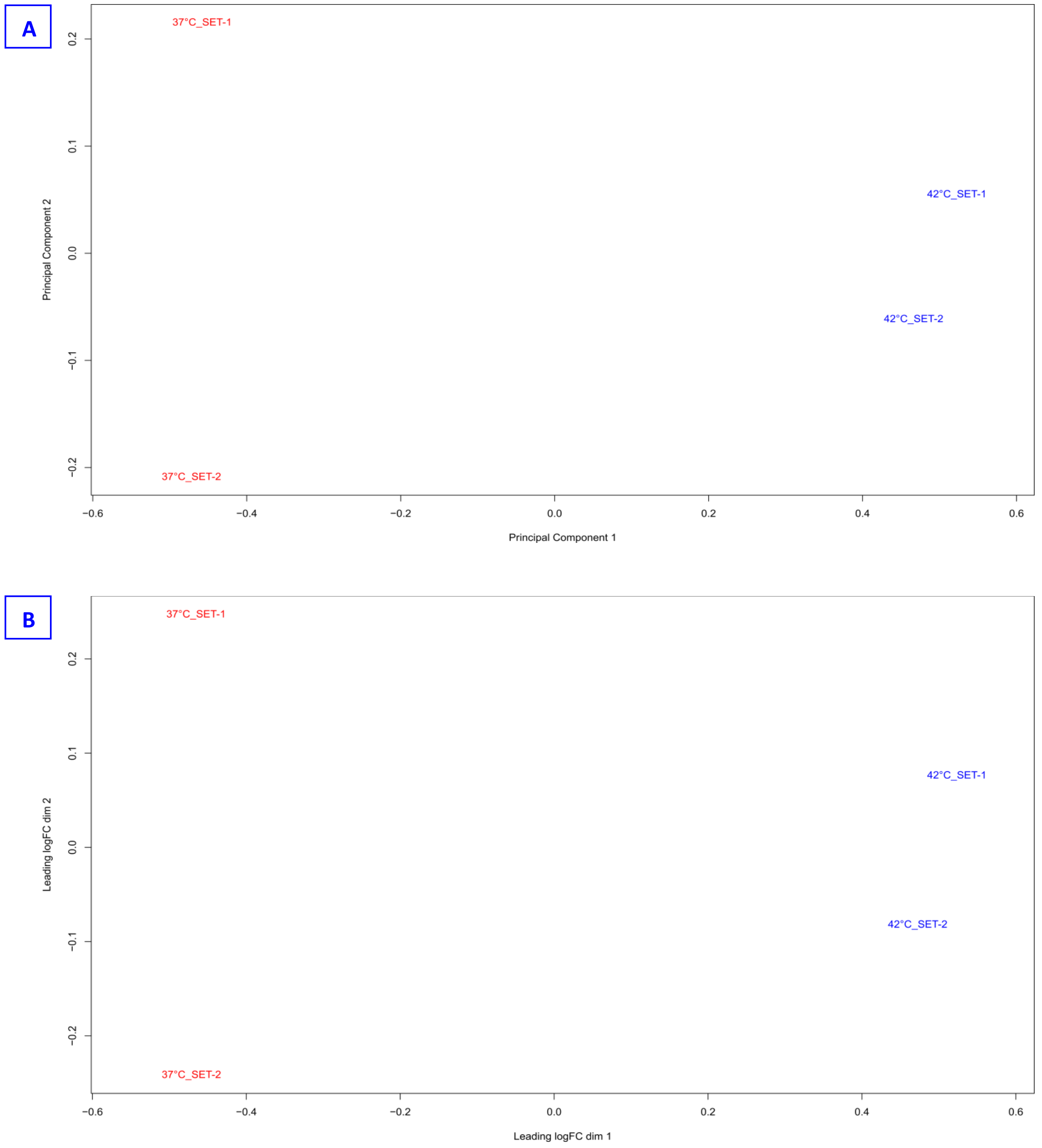

Supplementary Fig.S5

A: MDS plot with top 500 genes with the highest standard deviation in expression between samples.

B: MDS plot with top 500 genes with the highest fold change in expression between samples. The distance between each pair of samples is the root-mean-square deviation (Euclidean distance) for the top genes. Distances on the plot can be interpreted as leading log2-fold-change, meaning the typical (root-mean-square) log2-fold-change between the samples for the genes that distinguish those samples.

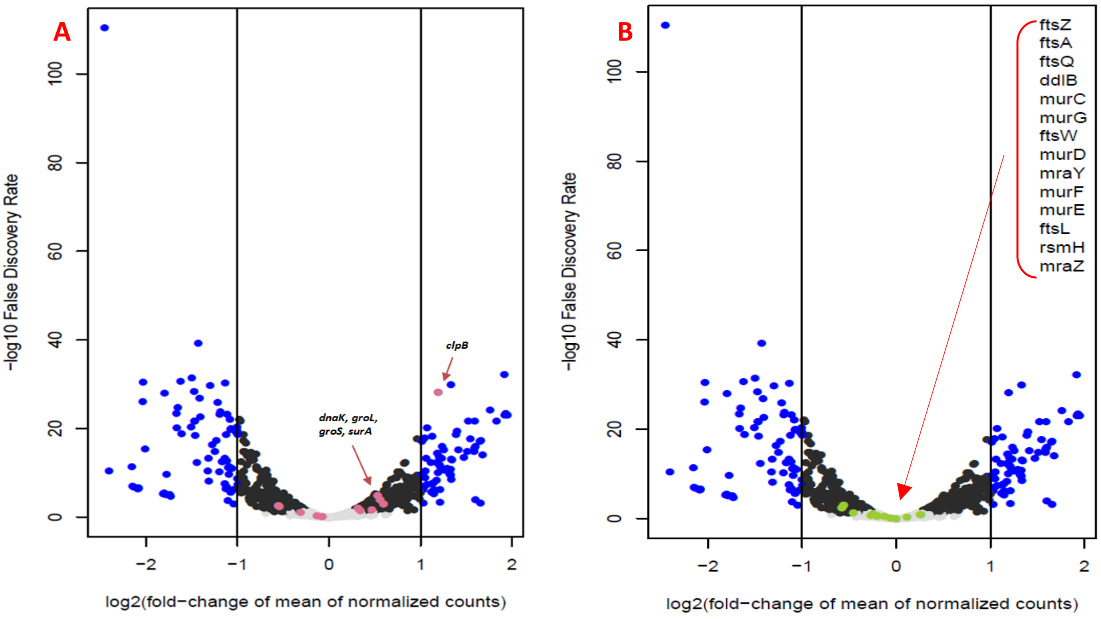

Supplementary Fig.S6: Volcano plot of differentially expressed genes

(A) chaperon proteins, (B) division cell wall (dcw) gene cluster

Gray = genes that do not change in significantly expression; black = genes with expression values associated with FDR-corrected p-values < 0.05; blue = genes expression values associated with with FDR-corrected p-value < 0.05 and linear fold change between 37°C and 42°C higher than 2.

Reference genome: *H. influenzae* Rd KW20 (NC_000907.1)	Gene	Function	GE47 strain - Growth at 37°C		GE47 strain - Growth at 42°C		Statistical analysis			
			SET-1	SET-2	SET-1	SET-2				
			Total reads= **12471697** Mapped reads (**79.9%**)= 9958912	Total reads= **12706187** Mapped reads (**80.2%**)= 15852996	Total reads= **13245098** Mapped reads (**81.9%**)= 16172459	Total reads= **13125243** Mapped reads (**82.1%**)= 15994253	**log2 (Fold change)**	**logCPM**	***P* Value**	**FDR**
HI0002	-	Long-chain-fatty-acid--CoA ligase FadD15	5494	8181	20023	18520	1.13	9.98	1.9E-20	6.4E-19
HI0039	mreD	Rod shape-determining protein MreD	810	829	2514	2396	1.19	6.99	7.4E-12	9.9E-11
HI0040	-	hypothetical protein	4161	4470	11868	11032	1.01	9.26	1.4E-11	1.8E-10
HI0041	xthA	Exodeoxyribonuclease III	5722	6692	17412	16048	1.04	9.80	3.3E-20	1.1E-18
HI0042	rluA_1	Ribosomal large subunit pseudouridine synthase A	2872	3887	11769	11112	1.38	9.15	6.9E-21	2.5E-19
HI0043	-	hypothetical protein	4324	5562	15100	15091	1.23	9.59	3.4E-18	9.5E-17
HI10044.1 (HI0044.1)	-	tRNA-Ser	56	63	211	181	1.33	3.35	3.0E-10	3.3E-09
HI0070	recN	DNA repair protein RecN	5347	6631	20465	18782	1.33	9.94	4.8E-33	1.0E-30
HI0075	nrdD	Anaerobic ribonucleoside-triphosphate reductase	820	1048	2561	2467	1.05	7.07	1.0E-10	1.2E-09
HI0086.1	-	tRNA-Cys	1348	1664	6129	5126	1.52	8.09	4.2E-24	2.2E-22
HI0086.2	-	tRNA-Gly	92	148	483	457	1.60	4.52	4.4E-18	1.2E-16
HI0086.3	-	tRNA-Leu	1969	1781	8190	6745	1.59	8.48	8.5E-18	2.3E-16
HI0086.4	-	tRNA-Lys	294	258	965	729	1.21	5.46	6.0E-08	4.8E-07
HI0088	thrB	Homoserine kinase	5236	6948	3648	3775	-1.09	8.70	2.3E-13	3.7E-12
HI0089	thrA	Bifunctional aspartokinase/homoserine dehydrogenase 1	14718	23134	9572	11138	-1.23	10.28	1.3E-19	3.9E-18
HI0092	gntP	High-affinity gluconate transporter	1853	2704	1201	1360	-1.20	7.24	4.0E-12	5.6E-11
HI0113.3 (HI_r02)	-	23S ribosomal RNA	10209	3970	2113	2498	-2.09	8.76	4.7E-08	3.8E-07
HI1003.5 (HI_r03)	-	16S ribosomal RNA	2302	995	703	597	-1.80	6.70	5.8E-07	3.8E-06
HI0113.6	-	tRNA-Pro	73	63	200	188	1.12	3.40	2.5E-07	1.8E-06
HI0113.7	-	tRNA-His	172	174	848	768	1.83	5.24	4.2E-24	2.2E-22
HI0122	metC	Cystathionine beta-lyase MetC	4098	7555	2509	3449	-1.32	8.54	5.9E-10	6.2E-09
HI0123.1	-	tRNA-Gly	111	141	505	429	1.51	4.53	6.8E-17	1.6E-15

HI0123.2	-	tRNA-Leu	1992	1740	8030	6800	1.58	8.47	5.7E-17	1.4E-15
HI0136.1	-	tRNA-Asp	4	15	34	41	1.60	1.03	2.5E-05	1.2E-04
HI0139	**ompP2**	Outer membrane protein P2 OmpP2	116878	146711	78732	77408	-1.14	13.13	2.4E-33	5.7E-31
HI0157	fabH	3-oxoacyl-[acyl-carrier-protein] synthase 3	6643	7470	19306	18056	1.01	9.97	2.7E-19	7.9E-18
HI0189	gdhA	NADP-specific glutamate dehydrogenase	3216	5263	1538	1894	-1.66	8.00	1.3E-22	6.0E-21
HI0220.4 (HI_r05)	-	23S ribosomal RNA	10275	4070	2126	2388	-2.13	8.77	1.4E-08	1.2E-07
HI0220.6	-	tRNA-Glu	6	8	32	26	1.65	0.68	1.3E-04	5.3E-04
HI0220.5 (HI_r06)	-	16S ribosomal RNA	2402	984	684	643	-1.81	6.74	1.0E-06	6.3E-06
HI0223	-	rarD protein, putative	516	527	1395	1423	1.04	6.24	8.8E-09	8.1E-08
HI0255	dapA	4-hydroxy-tetrahydrodipicolinate synthase	13912	17654	9090	8554	-1.22	10.04	9.8E-29	1.1E-26
HI0256	-	hypothetical protein	6926	7004	4570	4016	-1.10	8.92	1.5E-11	1.9E-10
HI0274.1	-	tRNA-Val	102	105	298	314	1.17	4.02	4.7E-10	5.0E-09
HI0274.2	-	tRNA-Val	98	101	283	292	1.14	3.94	1.9E-09	1.9E-08
HI0274.3	-	tRNA-Val	98	93	295	334	1.33	4.02	2.5E-11	3.1E-10
HI0274.4	-	tRNA-Val	93	93	283	327	1.32	3.98	1.7E-11	2.2E-10
HI0287	mtr	Tryptophan-specific transport protein	852	1072	2834	2948	1.21	7.22	1.1E-13	1.8E-12
HI0319	cmoA	tRNA (cmo5U34)-methyltransferase	2483	2854	7547	6937	1.05	8.59	2.5E-12	3.5E-11
HI0325	-	Na+/H+ antiporter family protein	9846	11033	6610	5587	-1.17	9.47	1.7E-14	3.1E-13
HI0331	-	hypothetical protein	373	550	1432	1424	1.26	6.19	1.9E-12	2.8E-11
HI0332	recO	DNA repair protein RecO	622	718	2496	2607	1.54	6.95	5.4E-20	1.7E-18
HI0333	rlmD	23S rRNA (uracil(1939)-C(5))-methyltransferase RlmD	673	798	2642	2469	1.41	6.98	2.9E-17	7.5E-16
HI0380.2	-	tRNA-Lys	289	265	922	707	1.15	5.42	9.7E-08	7.5E-07
HI0438	comB	competence protein B	178	164	110	104	-1.07	3.57	2.4E-07	1.7E-06
HI0443	recR	Recombination protein RecR	1812	2089	5488	6015	1.17	8.22	2.7E-14	4.7E-13
HI0444	topB	DNA topoisomerase 3	1389	1779	7528	7976	1.91	8.46	9.9E-36	5.5E-33
HI0465	serA	D-3-phosphoglycerate dehydrogenase	5739	7735	4065	3708	-1.17	8.82	2.0E-14	3.5E-13
HI0548	infA	Translation initiation factor IF-1	1077	1235	3466	3430	1.19	7.48	5.0E-14	8.3E-13
HI0561	-	OPT oligopeptide transporter protein	11630	17660	6995	7137	-1.42	9.86	1.2E-29	1.6E-27
HI0583	cpdB	2',3'-cyclic-nucleotide 2'-phosphodiesterase/3'-nucleotidase precursor	27355	31084	19020	19206	-1.00	11.01	5.4E-21	2.0E-19
HI0584	iaaH	Indole-3-acetyl-aspartic acid hydrolase	7380	8980	4507	4281	-1.28	9.08	1.9E-18	5.3E-17

HI0595	arcC1	Carbamate kinase 1	5386	7570	1843	2244	-2.04	8.55	1.2E-33	3.4E-31
HI0596	arcB_3	Ornithine carbamoyltransferase, catabolic	5236	7670	1742	2307	-2.04	8.54	5.7E-29	6.9E-27
HI0601.2 (HI_r07)	-	16S ribosomal RNA	2389	958	712	678	-1.73	6.74	3.9E-06	2.2E-05
HI0601.6	-	tRNA-Ile	120	140	490	579	1.66	4.69	1.4E-19	4.4E-18
HI0601.7	-	tRNA-Ala	80	114	477	482	1.93	4.48	4.6E-26	3.8E-24
HI0601.3 (HI_r08)	-	23S ribosomal RNA	10484	4141	2187	2365	-2.15	8.79	1.1E-08	9.7E-08
HI0609.1	-	tRNA-Pro	71	52	174	185	1.14	3.28	9.9E-07	6.2E-06
HI0621.2 (HI_r10)	-	16S ribosomal RNA	2440	997	726	650	-1.78	6.77	1.5E-06	9.1E-06
HI0621.3 (HI_r11)	-	23S ribosomal RNA	10339	4054	2238	2423	-2.09	8.78	2.8E-08	2.4E-07
HI0630	rseB	Sigma-E factor regulatory protein RseB precursor	3829	3375	12334	11009	1.29	9.21	6.9E-13	1.0E-11
HI0642.2	-	tRNA-Arg	1356	1632	4330	3799	1.06	7.76	2.8E-15	5.6E-14
HI0648	mdaB	Modulator of drug activity B	3707	5746	2151	1831	-1.61	8.18	3.8E-21	1.5E-19
HI0683	glpC	Anaerobic glycerol-3-phosphate dehydrogenase subunit C	1319	2078	5818	5262	1.34	8.12	6.2E-15	1.2E-13
HI0684	glpB	Anaerobic glycerol-3-phosphate dehydrogenase subunit B	1138	1748	4900	4902	1.40	7.93	1.1E-21	4.5E-20
HI0685	glpA	Anaerobic glycerol-3-phosphate dehydrogenase subunit A	1572	2274	5829	5830	1.23	8.22	2.5E-15	5.0E-14
HI0689	glpQ	Glycerophosphoryl diester phosphodiesterase precursor	37728	41704	20958	21348	-1.30	11.36	1.1E-32	2.1E-30
HI0693	hel	Lipoprotein E precursor	25668	31158	16726	17520	-1.11	10.92	7.7E-26	5.9E-24
HI0723.1 (HI_r13)	-	16S ribosomal RNA	2305	989	725	625	-1.75	6.71	1.3E-06	7.8E-06
HI0723.2	-	tRNA-Ile	138	140	473	537	1.47	4.65	1.4E-15	2.9E-14
HI0723.5	-	tRNA-Ala	85	101	451	466	1.92	4.42	1.2E-25	9.0E-24
HI0723.3 (HI_r14)	-	23S ribosomal RNA	10341	3997	2203	2439	-2.09	8.77	4.0E-08	3.3E-07
HI0749	lexA	LexA repressor	4203	4766	14432	12528	1.20	9.44	1.1E-15	2.3E-14
HI0761.1	-	tRNA-Phe	17	19	56	53	1.21	1.62	1.1E-04	4.7E-04
HI0761.2	-	tRNA-Asn	74	82	256	252	1.32	3.72	1.1E-11	1.4E-10
HI0775	cynR_1	HTH-type transcriptional regulator CynR	1474	1842	853	875	-1.32	6.76	2.9E-15	5.7E-14
HI0779	rplW / rpl23	50S ribosomal protein L23	34471	37692	23149	21874	-1.07	11.29	2.1E-22	9.5E-21
HI0781	rpsS19	30S ribosomal protein S19	30786	36301	22110	21318	-1.01	11.20	2.8E-22	1.2E-20
HI0818	galM	Aldose 1-epimerase	2070	3248	11696	11430	1.76	9.07	6.8E-27	6.7E-25
HI0819	galK	Galactokinase	2121	3135	10534	9905	1.59	8.93	3.9E-24	2.1E-22
HI0822	mglB	D-galactose-binding periplasmic protein precursor	31743	40153	23879	22643	-1.01	11.29	9.2E-23	4.5E-21

HI0848	trmA	tRNA/tmRNA (uracil-C(5))-methyltransferase	1752	1800	5548	5308	1.22	8.12	2.0E-13	3.3E-12
HI0853	hbpA_1 / dppA	Heme-binding protein A precursor	17907	22631	13062	13226	-1.01	10.47	8.7E-22	3.6E-20
HI0859	clpB	Chaperone protein ClpB	28409	38267	98886	98948	1.19	12.32	3.5E-31	5.4E-29
HI0875	pepB_2	Peptidase B	720	1039	456	591	-1.12	5.89	1.7E-08	1.5E-07
HI0974.1	-	hypothetical protein	744	756	459	437	-1.14	5.68	2.5E-09	2.5E-08
HI0975	panF	Sodium/pantothenate symporter	15592	19325	10955	10470	-1.09	10.23	1.6E-24	1.0E-22
HI0998	rpmH / rpL34	50S ribosomal protein L34	623	599	1670	1714	1.07	6.49	3.9E-09	3.7E-08
HI0999	rnpA	Ribonuclease P protein component	4668	4370	16039	14083	1.33	9.56	3.8E-15	7.4E-14
HI1000	yidD	Putative membrane protein insertion efficiency factor	1752	1386	7160	6218	1.67	8.30	3.9E-16	8.6E-15
HI1001	yidC	Membrane protein insertase YidC	18664	22126	59683	52236	1.07	11.53	1.4E-22	6.4E-21
HI1053	ahpD	Alkyl hydroperoxide reductase AhpD	1252	1529	889	923	-1.00	6.61	3.5E-09	3.3E-08
HI1120	oppF	Oligopeptide transport ATP-binding protein OppF	10233	13660	5448	5723	-1.47	9.56	3.4E-24	2.0E-22
HI1121	oppD_1	Oligopeptide transport ATP-binding protein OppD	7604	10316	5271	5419	-1.12	9.25	1.0E-14	1.9E-13
HI1122	oppC	Oligopeptide transport system permease protein OppC	7667	9825	5011	5295	-1.14	9.21	2.2E-15	4.4E-14
HI1123	oppB	Oligopeptide transport system permease protein OppB	10472	11040	4561	4426	-1.65	9.38	1.4E-27	1.5E-25
HI1124	oppA_2	Periplasmic oligopeptide-binding protein precursor	37004	46365	9816	9977	-2.46	11.17	2E-114	4E-111
HI1125	talB	Transaldolase B	18601	22770	12703	10832	-1.20	10.44	6.0E-26	4.8E-24
HI1167	serC	Phosphoserine aminotransferase	5321	6839	3220	3423	-1.25	8.65	5.3E-17	1.3E-15
HI1176	-	hypothetical protein	82	104	317	291	1.33	3.97	8.5E-13	1.3E-11
HI1177	artM	Arginine ABC transporter permease protein ArtM	339	461	1181	937	1.03	5.83	6.7E-08	5.3E-07
HI1180	artP	Arginine transport ATP-binding protein ArtP	275	344	958	800	1.12	5.54	2.9E-09	2.9E-08
HI1197	sucD	Succinyl-CoA ligase [ADP-forming] subunit alpha	3245	7009	2255	3752	-1.11	8.41	4.1E-05	1.8E-04
HI1217	-	putative TonB-dependent receptor precursor	12722	16950	8766	8109	-1.19	9.96	1.4E-26	1.3E-24
HI1225	-	translation initiation factor Sui1	1371	2216	861	977	-1.33	6.85	2.0E-12	2.9E-11
HI1247.1	-	tRNA-Asn	56	64	183	163	1.14	3.23	9.0E-08	7.0E-07
HI1359	glgC	Glucose-1-phosphate adenylyltransferase	5248	9730	4290	4963	-1.05	8.99	7.0E-08	5.5E-07
HI1360	glgA	Glycogen synthase	4509	8276	3257	4463	-1.08	8.75	3.1E-07	2.1E-06
HI1362	pntA	NAD(P) transhydrogenase subunit alpha	33960	49356	23090	26242	-1.12	11.46	7.5E-21	2.6E-19
HI1363	pntB	NAD(P) transhydrogenase subunit beta	25237	34717	17639	19191	-1.08	11.00	3.4E-22	1.4E-20
HI1379	phoB	Phosphate regulon transcriptional regulatory protein PhoB	10414	10475	7247	6487	-1.00	9.53	1.6E-10	1.8E-09

HI1380	pstB	Phosphate import ATP-binding protein PstB	9812	11190	5030	4905	-1.47	9.39	3.9E-24	2.1E-22
HI1381	pstA	Phosphate transport system permease protein PstA	11511	16809	6184	6949	-1.48	9.80	2.9E-31	4.9E-29
HI1382	pstC	Phosphate transport system permease protein PstC	19003	26948	9759	11231	-1.50	10.49	1.0E-34	4.4E-32
HI1383	pstS	Phosphate-binding protein PstS precursor	31430	46702	14672	17950	-1.63	11.22	6.7E-34	2.3E-31
HI1389.1	trpC	yadA	3065	3869	8377	10120	1.04	8.95	6.0E-11	7.2E-10
HI1403	-	Phage tail fiber repeat protein	203	360	167	163	-1.13	4.23	3.8E-08	3.1E-07
HI1405	-	hypothetical protein	42	60	29	35	-1.05	1.84	2.3E-04	8.7E-04
HI1424.1	-	tRNA-Leu	27	30	77	75	1.03	2.12	1.7E-04	6.6E-04
HI1530	gltS	Sodium/glutamate symport carrier protein	30992	37971	16818	16506	-1.43	11.11	6.2E-43	5.2E-40
HI1545	sstT	Serine/threonine transporter SstT	5471	7082	3881	3937	-1.06	8.76	6.3E-13	9.7E-12
HI1546	-	DNA polymerase V subunit UmuD	549	551	1574	1358	1.02	6.30	3.7E-08	3.1E-07
HI1601	-	hypothetical protein	1128	2449	543	775	-1.78	6.71	1.9E-11	2.4E-10
HI1603	-	Phosphate transport regulator	1624	1685	4809	5044	1.18	8.00	1.5E-16	3.4E-15
HI1617	aspC	Aspartate aminotransferase	3125	4219	1756	1831	-1.41	7.88	2.8E-25	1.9E-23
HI1632	lysC	Lysine-sensitive aspartokinase 3	6246	6826	2407	2514	-1.80	8.63	7.9E-31	1.1E-28
HI1702	metE	5-methyltetrahydropteroyltriglutamate-- homocysteine methyltransferase	16947	20126	11863	12007	-1.02	10.34	1.4E-21	5.3E-20
HI1707	qseC	Sensor protein QseC	4609	6296	2542	2426	-1.51	8.42	1.1E-22	5.1E-21
HI1708	qseB	Transcriptional regulatory protein QseB	3916	5421	2012	1793	-1.67	8.15	3.8E-26	3.3E-24
HI1709	-	hypothetical protein	8401	11751	5183	4275	-1.46	9.32	9.2E-21	3.2E-19
HI1725	**pbp1B (ponB)**	Penicillin-binding protein 1B	2805	3694	9918	10162	1.25	9.00	3.0E-17	7.5E-16
HI1728	mntH	Divalent metal cation transporter MntH	2454	4339	1371	1816	-1.45	7.73	2.3E-14	4.1E-13
HI1729	-	LamB/YcsF family protein	672	1410	219	371	-2.16	5.85	2.2E-13	3.5E-12
HI1730	kipA	KipI antagonist	903	2168	242	485	-2.41	6.36	2.0E-12	2.9E-11
HI1731	kipI	Kinase A inhibitor	770	1466	310	396	-2.01	5.99	1.8E-17	4.7E-16
HI1733	rnb	Exoribonuclease 2	3123	3841	9658	9924	1.11	9.01	3.1E-14	5.2E-13
HI1739.6 (HI_r17)	-	23S ribosomal RNA	10053	3975	2195	2377	-2.08	8.74	2.6E-08	2.3E-07
HI1739.5	-	tRNA-Ala	84	96	442	465	1.95	4.40	1.3E-25	9.2E-24
HI1739.4	-	tRNA-Ile	134	138	517	598	1.64	4.75	3.2E-19	9.2E-18
HI1739.3 (HI_r18)	-	16S ribosomal RNA	2327	996	723	641	-1.75	6.73	1.3E-06	7.7E-06

Supplementary Table S4: Summary statistics of RNA-seq profiles between 37°C and 42°C of the 141 differentially expressed genes with a |log2(fold change)| >1, including 67 up-regulated and 74 down-regulated.

LogCPM = Logarithm of counts per million reads

FDR = False discovery rat
